# The polymorphisms of the *MMP-1* and the *MMP-3* genes and the risk of pelvic organ prolapse

**DOI:** 10.1007/s00192-012-1970-1

**Published:** 2012-10-30

**Authors:** Paweł Skorupski, Katarzyna Jankiewicz, Paweł Miotła, Małgorzata Marczak, Beata Kulik-Rechberger, Tomasz Rechberger

**Affiliations:** 1 2nd Department of Gynecology, Medical University of Lublin, Jaczewskiego 8, 20-954 Lublin, Poland; 2 Department of Genetics and Microbiology, University of Maria Curie-Skłodowska, Lublin, Poland; 3 Department of Pediatric Propedeutics, Medical University of Lublin, Lublin, Poland

**Keywords:** Pelvic organ prolapse, Polymorphism, *MMP-1*, *MMP-3*

## Abstract

**Introduction and hypothesis:**

To investigate the associations between single nucleotide polymorphism (SNP) type 1G/2G at position −1607/−1608 of the matrix metalloproteinase *(MMP)-1* gene and SNP type 5A/6A at position −1612/-1617 of the *MMP-3* gene and the development of pelvic organ prolapse (POP) in women.

**Methods:**

133 patients with symptomatic POP were included in the study group. The control group consisted of 132 women with a normal pelvic floor. 1G/2G *MMP-1* and 5A/6A *MMP-3* SNPs were determined by polymerase chain reaction (PCR) and restriction fragments length polymorphism analysis.

**Results:**

When estimated individually none of the investigated SNPs were associated with POP. The combined *MMP-1/MMP-3* SNP analysis showed that the following polymorphic pairs were overrepresented in women with POP: 1G/2G −5A/6A, 2G/2G −5A/6A, 2G/2G −5A/5A, 1G/1G −6A/6A, *p* = 0.005.

**Conclusions:**

The combined effect of −1607/−1608 *MMP-1* and −1612/−1617 *MMP-3* SNPs may contribute to the development of POP in some women.

## Introduction

Pelvic organ prolapse (POP) affects 10–40% of older women [1]. Approximately 2% of women suffer from severe disturbances of the pelvic floor and finally become candidates for surgical repair [2]. The exact causes of POP are not known, but this condition could be linked to disturbances in connective tissue metabolism. The main structural protein of connective tissue is type I collagen—a heterotrimer comprising two α-1(I) and single α-2(I) chains encoded by the genes *COL1A1* and *COL1A2* respectively [3]. The important physiological role of this protein is to provide support for the pelvic floor structures. Metalloproteinases (MMPs) are enzymes capable of degrading collagens and other extracellular matrix (ECM) components. *MMP-1* plays a major role in the collagen type I degradation, whereas *MMP-3* is able to activate other MMPs, including *MMP-1* [4]

There is also evidence suggesting that POP might be hereditary. A study by Jack et al. [5] showed that the risk of POP is 5-fold higher in siblings of women with advanced disease. Analysis of monozygotic and dizygotic twins indicates that both genetic and environmental risk factors contribute to the etiology of POP and stress urinary incontinence (SUI) [6]. Dietz et al. [7], in a study of nulliparous young women, demonstrated the heritability of bladder neck mobility. Molecular mechanisms behind the aforementioned findings remain unknown, but may involve single nucleotide polymorphisms (SNPs) in the regulatory areas of the genes encoding proteins relevant to connective tissue function. These polymorphisms change patterns of collagens and *MMPs* genes expression. Some of the SNPs in genes encoding ECM proteins are associated with bone brittleness [8], heart conditions [9] or cancer susceptibility [10]. The development of SUI, which shares many risk factors with POP, is also associated with SNP in the gene encoding α-1 chain of type I collagen [11].

Genes encoding collagen-degrading enzymes—matrix metalloproteinase type 1 (collagenase-1) and matrix metalloproteinase type 3 (stromelysin)—are located on the long arm of chromosome 11 [12]. SNP due to the insertion of the extra guanine (G) base at position −1607/−1608 upstream from the start of transcription creates a binding site for Ets family transcription factors. The binding of Ets to the DNA strand upregulates *MMP-1* transcription and possibly increases the tissue activity of this enzyme.

The main substrates for *MMP-3* are collagens type II, III, and IV [13]. However, the substrate specificity of *MMP-3* encompasses several other collagens, fibronectin, gelatin, and elastin [14]. Another important feature of the *MMP-3* is the ability to activate other members of the MMP family [15]. The insertion of the adenosine (A) base in the promoter of the *MMP-3* gene, at position −1612/−1617 upstream from the start of transcription, creates a polymonomeric run of six adenosines (6A allele), while the other variant has five adenosines (5A allele). The presence of the 6A allele enables binding of the repressor ZBP-89 that downregulates the expression of the *MMP-3* gene [16]. We hypothesized that modifications of ECM metabolism caused by polymorphisms of the *MMP* genes could contribute to the development of POP. Therefore, the aim of the study was to estimate the associations between the SNP at position −1607/−1608 of the *MMP-1* gene and the SNP at position −1612/−1617 of the *MMP-3* gene, and between the combinations of the genotypes created by the above-mentioned SNPs and the risk of pelvic organ prolapse.

## Materials and methods

The study was approved by the Institutional Ethics Committee. All patients gave their informed consent. Recruitment took place from 2007 to 2008. All patients were referred from outpatient settings and were asked to participate in this study after admission. One hundred and thirty-three women with symptomatic pelvic organ prolapse (grades II, III, and IV; POPQ) were included into the study group [17]. These patients were subjected to pelvic floor repair procedures. Anterior and posterior vaginal wall defects were treated by the insertion of anterior mesh implant or posterior mesh implant, respectively. For combined anterior/posterior defects anterior and posterior implants were placed during a single surgical procedure. Besides mesh surgery some patients required colpoperineoplasty for the perineal body reinforcement.

The control group consisted of 132 women without significant POP (grades 0, I; POPQ). The vast majority of these patients were admitted with uterine myomas and subsequently underwent total abdominal hysterectomy or supracervical abdominal hysterectomy. The rest of the control group consisted of patients with dysfunctional uterine bleeding (DUB). They were subjected to endometrial biopsy or in a few cases to dilatation and curettage. All subjects in the study and the control group were assessed with the same diagnostic work-up that included measurement of the vital signs, body weight and height for BMI calculation, physical examination with assessment of POP, bimanual gynecological examination, and routine pre-operative laboratory tests. Patients with connective tissue and autoimmune diseases, joint and bone diseases, cancer, chronic inflammation (e.g., Crohn’s disease, ulcerative colitis) or any serious or life-threatening conditions were excluded from the study.

### Laboratory procedures

Blood samples were taken prior to surgery into tubes containing anticoagulant EDTA. Genomic DNA was extracted from whole-blood leukocytes using a commercially available kit (GenomicPrep Blood DNA Isolation Kit, Amersham Biosciences, USA). DNA was stored at −20°C until used. Determination of *MMP-1* and *MMP-3* polymorphisms was carried out using polymerase chain reaction (PCR) and restriction fragments length polymorphism (RFLP) analysis.

### *MMP-1* polymorphism

**Fig. 1:**
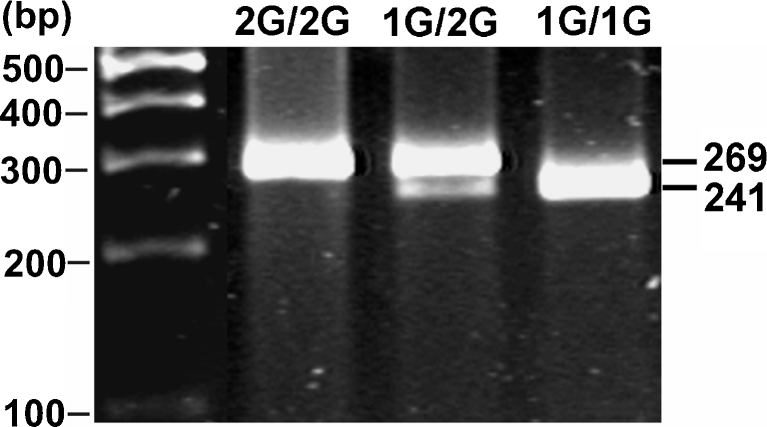
Analysis of *MMP-1* 1G/2G polymorphism. Polymerase chain reaction (PCR) products were digested with *Alu*I restriction endonuclease and separated on 3% agarose gel. The ethidium bromide-stained gel was visualized using UV transillumination. A single band of 269 bp corresponds to 2G/2G homozygote, two bands of 241 and 28 bp to 1G/1G homozygote, and three bands of 269, 241, and 28 bp to heterozygote 1G/2G (the smallest band [28 bp] is not visible)

Polymerase chain reaction was performed (Biometra T personal Thermocycler, Whatman Biometra, Germany) using 40 ng of DNA per reaction. For amplification, the Taq DNA polymerase (Promega, Madison, WI, USA) and commercially obtained oligonucleotide primers; 5′-TGACTTTTAAAACATAGTCTATGTTCA′ (forward) and 5′-TCTTGGATTGATTTGAGATAAGTCATAGC-3′ (reverse) were used. The 40 amplification cycles were preceded by denaturation at 99°C for 7 min. Annealing was carried out at 52°C for 30 s, elongation at 72°C for 45 s, and denaturation 94°C for 30 s. A final primer extension was carried out at 72°C for 5 min. Reverse primer was designed to introduce restriction site for the enzyme *Alu*I (AGCT) by replacing T with a G at the second position close to 3′ end of the reverse primer [18]. The final recognition of the *MMP-1* polymorphism was based on analysis of RFLP. PCR products (10 μl) were digested with *Alu*I (Fermentas) (10 U, 37°C, 16 h), separated on 3% agarose gel, and stained with ethidium bromide (Fig. 1).

### *MMP-3* polymorphism

**Fig. 2:**
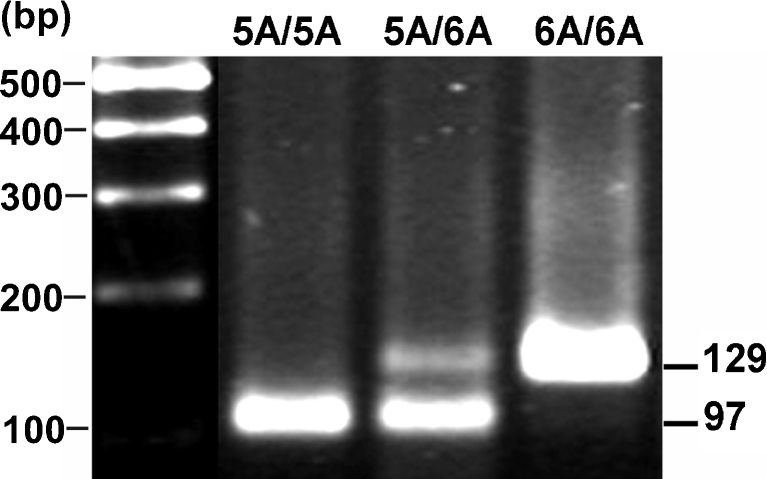
Analysis of the *MMP-3* 5A/6A polymorphism. PCR products were digested with *Tht*111 restriction endonuclease and separated on 3% agarose gel. The ethidium bromide-stained gel was visualized using UV transillumination. A single band of 129 bp corresponds to the 6A/6A homozygote, three bands of 129, 97 and 32 bp to the 5A/6A heterozygote and two bands of 97 and 32 bp to the 5A/5A homozygote (the smallest band [32 bp] is not visible)

Polymerase chain reaction was carried out using 40 ng DNA per well. The PCR primers were: 5′-GGTTCTCCATTCCTTTGATGGGGGGAAAgA-3′ (forward) and 5′-CTTCCTGGAATTTCACATCACTGCCACCACT-3′ (reverse). The conditions of PCR reactions were as follows: denaturation 5 min at 98°C followed by 40 cycles of annealing 30 s 58°C, elongation 30 s 72°C and denaturation 30 s 94°C with final elongation 5 min 72°C. PCR products (10 μl) were digested with *Tht*111 10 U/sample at 37°C, 16 h and separated on 3% agarose gel [19]. After staining with ethidium bromide identification of the type of polymorphism was carried out (Fig. 2).

### Statistical analysis

Power calculations were performed prior to the study, based on the data from the HapMap project regarding the allele frequency. We assumed that 130 patients in each group would have been enough to show the association between the polymorphisms investigated and POP. Pearson’s Chi-squared test was used for the comparison of the polymorphism frequencies between study and control patients. Because the distributions of age, parity, body mass index, and menopausal status were skewed, differences in means and medians were tested using the Mann–Whitney test. The statistical significance for deviations from the Hardy–Weinberg equilibrium were determined using Pearson’s Chi-squared test. All statistics were performed with Statistica v.6.1 (StatSoft, Tulsa, OK, USA).

## Results

The clinical characteristics of the patients are given in Table 1. The patients in the control group were younger than their counterparts in the study group. Not surprisingly, fewer patients in the control group had entered the menopause (Table 1).

**Table 1:** Characteristics of the study group and the control patients

	Control group (*n* = 132)	Study group (*n* = 133)	*p*
Age (years)	50.8	57.3	< 0.05
BMI (kg/m^2^)	27	27.7	ns
Parity (median)	2	2	ns
Cesarean section (median)	1	1,5	ns
Menopause (*n*)	58	79	< 0.05

### *MMP-1* alleles/genotypes analysis

The frequencies of the 1G/1G, 1G/2G, 2G/2G alleles were not consistent with the Hardy–Weinberg equilibrium. Table 2 displays the frequencies of the 1G and 2 G alleles and the *MMP-1* genotype distribution in the groups being investigated. We did not find any statistically significant differences between the study group and controls.

**Table 2:** Frequency of the 1G and 2G alleles and *MMP-1* genotype distribution in the groups being investigated

Group	*MMP-1* polymorphism (position −1607/−1608)						
	1G/1G	Percentage	1G/2G	Percentage	2G/2G	Percentage	Pearson’s Chi-squared test
Control (*n*=132)	45	34.1	54	40.9	33	25	χ^2^ = 0.07, *p* = 1
Study (*n*=133)	47	35.3	54	40.6	32	24.1	

### *MMP-3* alleles/genotypes analysis

The frequencies of the 5A/5A, 5A/6A, and 6A/6A alleles were not in Hardy–Weinberg equilibrium. Table 3 shows the frequency of the 5A and 6A alleles and *MMP-3* genotypes distribution in study subjects and controls. The statistically significant differences between the study and control groups were not found. The exact determination of SNPs in 7 POP samples was not possible because PCR-RFLPs repeatedly gave ambiguous results. These patients were not included in the analysis.

**Table 3:** The frequency of the 5A and 6A alleles and *MMP-3* genotype distribution in study subjects and controls

Group	*MMP-3* polymorphism (position −1612/−1617)						
	5A/5A	Percentage	5A/6A	Percentage	6A/6A	Percentage	Pearson’s Chi-squared test
Control (*n* =132)	34	25.8	79	59.9	19	14.4	χ^2^ = 0.6, *p* = 0.8
Study (*n* =126)	28	22.2	81	64.3	17	13.5	

### The combination of the *MMP-1* and *MMP-3* SNPs

Table 4 displays the combination of the *MMP-1* and *MMP-3* SNPs in the study and control groups. The comparison of the frequencies of the combination of the two genotypes, one for *MMP-1* and the other for *MMP-3*, showed that some of them were over-represented (1G/2G–5A/6A, 2G/2G–5A/6A, 2G/2G–5A/5A, 1G/1G–6A/6A, *p* = 0.005) in women with POP.

**Table 4:** Genotype combinations of the position −1607/−1608 *MMP-1* polymorphism and the position −1612/−1617 *MMP-3* polymorphism

Control group, freq. of polymorphisms (%)		*MMP-3*		
		5A/5A	5A/6A	6A/6A
*MMP-1*	1G/1G	39.5	58.1	2.3
	1G/2G	26.4	56.6	17
	2G/2G	6.2	68.7	25
Study group, freq. of polymorphisms (%)				
*MMP-1*	1G/1G	39.5	51.2	9.3^b^
	1G/2G	14^a^	68^b^	18
	2G/2G	10.3^b^	75.9^b^	13.8^a^

The results are the observed frequencies of the study (POP) patients and controls. Pearson’s Chi squared test = 28.6, *p* = 0.005,

^a^SNPs underrepresented in the study group

^b^SNPs overrepresented in the study group

There is also evidence that some combinations of genotypes appear with lesser frequency in women with POP (1G/2G–5A/5A, 2G/2G–6A/6A).

## Discussion

The rates of transcription of the *MMP* genes are influenced by SNPs in their regulatory elements. Studies show that the 2G/2G *MMP-1* polymorphism is linked to the aggressive phenotype and the process of metastasis formation in cancers [20, 21]. The elevated tissue *MMP-1* activity in carriers of 2G/2G genotype could be responsible for the degradation of fibrillar collagens and other components of ECM, thus facilitating the progress of the neoplastic disease. Other data suggest that the polymorphism-related modifications of expression of the *MMP-1* gene are also involved in the pathogenesis of idiopathic pulmonary fibrosis [22] and liver cirrhosis in HCV-infected patients [23]. Both idiopathic pulmonary fibrosis and liver cirrhosis were associated with the 2G/2G *MMP-1* variant, but unlike cancer the effect of this polymorphism is increased collagen content in the organs involved. Nonetheless, these results further support the role of the 1G/2G *MMP-1* SNP in the regulation of tissue collagen content.

The SNP at position –1612/–1617 forms clinically important variants of the *MMP-3* gene. 5A/5A individuals have the greatest transcription of the *MMP-3* gene, whereas those with 6A/6A SNP have the lowest level of the transcription [16]. The increased *MMP-3* activity in colorectal cancer suggests that this enzyme might play a role in the progression of the disease [24]. In contrast, the reduced activity of *MMP-3* in 6A/6A individuals with coronary heart disease contributed to the increased risk of restenosis, perhaps because of the failure of connective tissue remodeling [25].

We speculated that similar mechanisms related to the 1G/2G *MMP-1* and 5A/6A *MMP-3* SNPs might exist within the pelvic floor connective tissue in women with clinically significant pelvic organ prolapse. Theoretically, subjects with 2G/2G *MMP-1* polymorphism could have elevated *MMP-1* activity and the increased rate of collagen type I degradation. Also, the higher connective tissue *MMP-3* activity in 5A/5A subjects may be responsible for accelerating the breakdown of collagen and other ECM components. On clinical grounds, both SNPs could result in the elevation of the risk of POP. Our results did not support this view because, when considered separately, either the 1G/2G *MMP-1* or the 5A/6A *MMP-3* SNP is not associated with POP development. The other explanation of our results is that the association is simply too small to detect. This is in contrast to the results of the study by Vishwajit et al. [26], where a link between the 2G/2G *MMP-1* polymorphism and the risk of POP and SUI was found. The explanation for this discrepancy could be the weak statistical power of this study with just 28 subjects enrolled. However, the results of the study by Ferrari et al. showed that the *MMP-1* polymorphism could be linked to the susceptibility to POP [27]. Interestingly, these associations did not exist for *MMP-3* and *MMP-9* polymorphisms.

The analysis of allelic pairs showed that on the one hand, patients with 2G/2G *MMP-1* and 5A/5A as well as 5A/6A *MMP-3* polymorphisms appear to be more prone to developing POP. Genotypes with the greatest transcription levels of the *MMP-1* and *MMP-3* genes carry the greatest risk of POP. Therefore, it is possible that to become clinically evident the biological effect of the 2G/2G *MMP-1* variant has to be enhanced by the 5A/5A or the 5A/6A *MMP-3* SNP. On the other hand, the combination of the 2G/2G *MMP-1* polymorphism and the 6A/6A *MMP-3* polymorphism appears to be associated with a normal pelvic floor. Thus, we can speculate that the simultaneous presence of the 6A/6A *MMP-3* and the 2G/2G *MMP-1* SNPs diminishes the rate of collagen type I degradation. Of course, the mechanisms behind this phenomenon have remained elusive, but a possible explanation of our findings may lie in the regulation of the tissue activity of the MMPs. In addition to cytokines and growth factors MMPs themselves also modulate their activity. *MMP-3* is able to activate proMMP-1, further augmenting the tissue activity of *MMP-1* [15]. This may explain why women carrying the 2G/2G–5A/5A and 2G/2G–5A/6A genotypes are at greater risk of having clinically significant POP. Also, the 6A/6A variant seemed to play a protective role in POP only in conjunction with the 2G/2G *MMP-1* genotype. This finding corresponds with data showing a lower risk of lung and breast cancers in carriers of this polymorphic variant [19, 28]. However, the same polymorphic variant can also exert deleterious effects. The lower tissue activity of *MMP-3* in 6A/6A patients alters the remodeling process of ECM in the direction of facilitating the progress of atherosclerosis [29].

It is likely that MMPs and other regulatory proteins not estimated in this study are involved in the development of POP. For example, in patients with recurrent hernia there are increased connective tissue levels of *MMP-1* and *MMP-13* mRNAs and the levels of the corresponding proteins [30]. Tissue inhibitors of metalloproteinases (TIMPs) are able to inhibit active forms of MMPs. Thus, the connective tissue contents of TIMPs are crucial for MMP activity in vivo. The example came from a gastric cancer study in which the diminished TIMP-2 expression related to the gene promoter polymorphism contributed to rapid dissemination and worse patient survival [31]. Since our study was limited to the *MMP-1* and* MMP-3* polymorphisms, the role of TIMPs in the development of pelvic floor defects was not assessed. Therefore, we cannot rule out that the interpretation of our results would be different if the determination of TIMPs polymorphisms had been carried out. Undoubtedly, the weakness of our study is that SNP distribution was not in Hardy–Weinberg equilibrium. This could be attributed to the non-random selection of control and study subjects or too small a number of participants. However, the latter issue is less likely because the number of women recruited is comparable to those of similar SNP studies. Also, the control and study groups did not fully match with regard to the demographic and the clinical characteristics. This issue was a consequence of the fact that after the exclusion of malignancies the vast majority of the remaining patients were treated for DUB, symptomatic myomas or typical ailments of the late menopause, e.g., POP, SUI. Another limitation is the weak statistical power of the study caused by the unexpected distribution of allele frequencies in our patients. This finding could be explained by the genetic differences within Caucasian populations.

Besides clinical and environmental factors the disturbances in the female pelvic floor may also be dependent on the polymorphisms of the genes encoding the ECM components. The results of our study showed that certain combinations of the 1G/2G *MMP-1* and the 5A/6A *MMP-3* promoter SNPs are more likely to occur in women with POP than in the population with a normal pelvic floor. This newly found association should be considered, along with prolonged hypoestrogenism, multiple vaginal births, pelvic floor surgery, as a genetic risk factor for the development of pelvic organ prolapse.
